# Deaf-Mutism

**Published:** 1895-03

**Authors:** 


*Deaf-Mutism.* By HOLGER MYGIND, M.D. Pp. vii., 300. London: F. J. REBMAN. 1894.

The author of this book pleads for indulgence to any shortcomings of language, on the ground that he is not an Englishman, but such indulgence is by no means necessary inasmuch as the language would do credit to any English writer. The book bristles with statistics which are, of course, drawn largely from Danish sources, but tables are also given from all countries of Europe as well as from America. Careful investigations are found here into the relative proportions of congenital and acquired deaf-mutism, one of the most curious features of which is the fact that the statement as to the numerical proportion between congenital and acquired deaf-mutism varies greatly in different countries. The author has carefully investigated the interesting questions of hereditary tendencies and the effects of consanguineous marriages, with both of which he deals in an eminently judicial spirit.

The vexed question of the best mode of educating deaf-mutes is very fairly summed up as follows: "There can no longer be any doubt that the method of instruction by which the deaf-mutes are taught to speak is superior to the method by which deaf-mutes are taught to communicate with their fellow-creatures by means of signs." But the author adds, that the question whether the oral method is the most serviceable for *all* deaf-mutes must be left to pedagogues.

The book should be carefully studied by all who are interested in the treatment or instruction of deaf-mutes.

